# Bilirubin ameliorates osteoarthritis via activating Nrf2/HO‐1 pathway and suppressing NF‐κB signalling

**DOI:** 10.1111/jcmm.18173

**Published:** 2024-03-17

**Authors:** Xinyu Zhao, Baiqun Duan, Jianing Wu, Lihui Huang, Sheng Dai, Jie Ding, Meng Sun, Xinlu Lin, Yiling Jiang, Tuyue Sun, Ruijie Lu, Huirong Huang, Guangyong Lin, Ruijie Chen, Qing Yao, Longfa Kou

**Affiliations:** ^1^ Wenzhou Municipal Key Laboratory of Pediatric Pharmacy, Department of Pharmacy The Second Affiliated Hospital and Yuying Children's Hospital of Wenzhou Medical University Wenzhou China; ^2^ Key Laboratory of Structural Malformations in Children of Zhejiang Province Wenzhou China; ^3^ Zhejiang Engineering Research Center for Innovation and Application of Intelligent Radiotherapy Technology Wenzhou China; ^4^ School of Pharmaceutical Sciences Wenzhou Medical University Wenzhou China; ^5^ Zhejiang‐Hong Kong Precision Theranostics of Thoracic Tumors Joint Laboratory Wenzhou China

**Keywords:** anti‐inflammation, antioxidant, bilirubin, Nrf2/HO‐1, osteoarthritis

## Abstract

Osteoarthritis (OA) is a chronic degenerative joint disease that affects worldwide. Oxidative stress plays a critical role in the chronic inflammation and OA progression. Scavenging overproduced reactive oxygen species (ROS) could be rational strategy for OA treatment. Bilirubin (BR) is a potent endogenous antioxidant that can scavenge various ROS and also exhibit anti‐inflammatory effects. However, whether BR could exert protection on chondrocytes for OA treatment has not yet been elucidated. Here, chondrocytes were exposed to hydrogen peroxide with or without BR treatment. The cell viability was assessed, and the intracellular ROS, inflammation cytokines were monitored to indicate the state of chondrocytes. In addition, BR was also tested on LPS‐treated Raw264.7 cells to test the anti‐inflammation property. An in vitro bimimic OA microenvironment was constructed by LPS‐treated Raw264.7 and chondrocytes, and BR also exert certain protection for chondrocytes by activating Nrf2/HO‐1 pathway and suppressing NF‐κB signalling. An ACLT‐induced OA model was constructed to test the in vivo therapeutic efficacy of BR. Compared to the clinical used HA, BR significantly reduced cartilage degeneration and delayed OA progression. Overall, our data shows that BR has a protective effect on chondrocytes and can delay OA progression caused by oxidative stress.

## 1 INTRODUCTION

Osteoarthritis (OA) is the most common joint disorder, affecting approximately 528 million people and causing pain and disability, particularly in the elderly but also in young individuals.^1^ The disease mainly occurs in the articular hyaline cartilage of load‐bearing joints.^2^ As the OA condition progresses, there is severe cartilage degeneration, subchondral bone thickening, narrowing of the joint space, formation of osteophytes or bone spurs and joint inflammation along with swelling and pain.^3^, ^4^ Current treatments are limited to symptom relief with anti‐inflammatory drugs and replacement arthroplasty which is referring to a replacement surgery for degenerated joints by replacing it with a prosthetic implant at the end stage of OA.^5^ Identifying effective candidate drugs for OA therapy is of great clinical significance.

Previous research has shown that excessive reactive oxygen species (ROS) and subsequent oxidative cell injury play critical roles in the development and progression of OA chondrocytes.^6^, ^7^ The increased ROS levels can lead to cartilage degradation in OA due to the disruption of metabolic and homeostatic processes caused by the excessive production of matrix‐degrading proteases, such as matrix metalloproteinases (MMPs) and aggrecanases, while also reducing the expression of anabolic COL2α.^8^ Moreover, high levels of ROS are also involved in multiple signalling pathways, leading to inflammation, exacerbation of metabolic disorders, apoptosis and eventually the OA development. Therefore, pharmacological modulation of the relevant signalling pathways may be a promising approach to alleviate OA.

Increasing evidence suggests that the nuclear factor erythroid 2 (Nrf2) plays an important role as a transcription factor that regulates the cellular defence in response to oxidative stress and exerts protective effects against OA.^9^ The Nrf2/HO‐1 pathway is a classic antioxidant signalling pathway that coordinated the expression of enzymes involved in the cellular antioxidant defence, like phase II detoxification enzymes, which are closely related to cell apoptosis. Activation of this pathway has been shown to relieve oxidative damage and apoptosis in cartilage.^10^ The pivotal role of NF‐κB activation in various inflammatory conditions, including OA, is well recognized, particularly in the initiation of pro‐inflammatory cytokines like IL‐1α, IL‐1β and IL‐6.^11^, ^12^ For example, upon stimulation by IL‐1β, the rapid degradation of IκBα leads to the release of multiple NF‐κB dimers, contributing to inflammatory responses.^13^ It is crucial to highlight the intricate interaction between ROS and NF‐κB signalling pathways. Following NF‐κB activation, inflammatory responses may cause an upsurge in intracellular ROS.^14^ Arra et al. demonstrated that NF‐κB activation in OA prompts a metabolic shift in chondrocytes towards aerobic glycolysis, inducing oxidative stress via lactate dehydrogenase A activity.^15^ Furthermore, ROS can modulate NF‐κB activity through multifaceted and simultaneous interactions, influencing its levels in diverse ways.^16^ Moreover, the activation of the Nrf2/HO‐1 signalling pathway shows promise in suppressing NF‐κB‐mediated effects, offering potential benefits for treating OA.^17^

Bilirubin (BR) is a metabolic end product of heme catabolism and an endogenous antioxidant with the potent scavenge capacity to scavenge free radicals.^18^ For many decades, BR was considered as a toxin due to its biological effect in physiological neonatal jaundice. Unconjugated BR is lipid soluble and easily get into the brain, because the blood–brain barrier in premature babies is not fully developed; and the BR will be deposited in the basal ganglia, inducing adverse effect on brain development. Until 1980s, it was the first time that Stocker et al. reported the beneficial role of BR on protecting from free radicals‐induced oxidative damage.^19^ In response to ROS, BR could be oxidized to biliverdin, while biliverdin would be translated back to BR by biliverdin reductase. This cycle, just like the glutathione‐oxidized glutathione cycle, could amplify the antioxidative property of BR. Following that, increasing evidence have suggest that BR with potent antioxidant and anti‐inflammatory property could protect against various oxidative stress in a wide range of diseases.^20^, ^21^, ^22^, ^23^, ^24^, ^25^, ^26^, ^27^ However, there is currently a lack of fundamental mechanism investigations into the potential antioxidative stress and anti‐inflammatory effects of BR on OA.

Building upon the above, we analysed the impact of BR on variations in oxidative stress, inflammatory responses and apoptosis and investigated the underlying mechanisms in H_2_O_2_‐stimulated chondrocytes. Additionally, we evaluated the effects of ACLT on mouse cartilage in an experimental OA model and assessed the prognostic implications of BR intervention in the ACLT group. This study aimed to investigate the potential antioxidant and chondroprotective effects of BR on OA, as well as its underlying mechanisms.

## 2 METHODS AND MATERIALS

### 2.1 Chemicals and reagents

Bilirubin (BR) was purchased from Macklin Biochemical Co., Ltd. (Shanghai, China). ROS assay kit, TUNEL assay kit, anti‐fluorescence quenching agent with DAPI, Annexin V‐FITC Apoptosis Detection Kit, BCA protein assay kit and Haematoxylin & Eosin staining kit were purchased from Beyotime Biotechnology Co. Ltd. (Shanghai, China). Lipopolysaccharide (LPS), methyl thiazolyl tetrazolium (MTT), EDTA decalcifying solution, Toluidine Blue O and Modified Safranine O‐Fast Green FCF Cartilage Stain Kit were obtained from Beijing Solarbio Science & Technology Co., Ltd. (Beijing, China). Prime script RT reagent kit was bought from Vanzyme Biotech (Nanjing, China) Normal goat serum, neutral balsam and bovine serum albumin (BSA) were from Solarbio science & technology (Beijing, China). Antibodies to β‐actin (AF7018) and were bought from Affinity Biosciences (Jiangsu China). Immunohistochemical antigen repair buffer and Horseradish peroxidase‐conjugated secondary antibody were purchased from Zhongshan Jinqiao Biological Technology Co., Ltd. (Beijing, China). Goat Anti‐Rabbit IgG, Peroxidase Conjugated, round coverslips, were purchased from Biosharp (Hefei, China). Cell culture dishes/plates and centrifuge tubes were obtained from NEST Biotechnology Co.Ltd.(Wuxi, China). Dulbecco's modified eagle medium (DMEM) was obtained from Gibco (Massachusetts, USA)0.1 × Phosphate Buffer solution (PBS) was obtained from Servicebio (Wuhan, China). qRT Master mix (RTQ‐100) was purchased from TOROIVD (Shanghai, China). Green Taq Mix (P131) was bought from Nanjing Vazyme Biotech Co, Ltd (Nanjing, China). GelRed Nucleic Acid Stain (41003) was purchased from Biotium (San Francisco, USA). HRP‐conjugated secondary antibody (goat anti‐mouse and goat anti‐rabbit) were purchased from Biosharp Biotechnology (Anhui, China).

### 2.2 Animals

Twenty‐four SD male rats, which were 6 weeks old, weight ranging from 180 g to 220 g were purchased from Wenzhou Medical University. All animal housing and experiments were conducted in accordance with the guidelines of the Animal Ethics Committee of Wenzhou Medical University and with approval. All animals were housed under SPF condition.

### 2.3 Cell lines

Rat chondrocytes were grown in DMEM/Hams F‐12 medium with 10% FBS and 1% penicillin /streptomycin. Raw 264.7 cells were grown in DMEM with 10% FBS and 1% Penicillin/Streptomycin. All cells were grown and maintained at 37°C in an atmosphere of 95% air and 5% CO_2_.

### 2.4 Cell viability assay

The methyl‐thiazolyl‐tetrazolium assay (MTT) was used to determine cell viability. Rat chondrocytes were seeded into 96‐well plates at a density of 1 × 10^4^ cells/well and cultured for 12 h. The cells were then treated by H_2_O_2_ and/or BR according to the experimental design. After appropriate treatment, cells were incubated with MTT solution (20 μL) for 4 h. The absorbance was determined with a microplate reader (Infinite M200 pro, TECAN, Switzerland) at 490 nm.

### 2.5 ROS measurement

DCFH‐DA fluorescent probe was used to detect the effect of BR on intracellular ROS levels in chondrocytes. After different treatments, chondrocytes were incubated with DCFH‐DA at a concentration of 10 μM for 20 min under dark. After PBS washing (3 times, 5 min each), the chondrocytes were then imaged under a confocal laser scanning microscope (NIKON). Quantitative analysis was performed by using Image J software.

### 2.6 TUNEL staining

TUNEL assay was used to detect apoptotic chondrocytes. Chondrocytes were treated with different drug treatments for 24 h in 6‐well plate. After PBS washing (3 times, 5 min each), the chondrocytes were fixed and then stained with TUNEL assay kit. After that, the cells were observed under a fluorescence microscope (BX53, OLYMPUS).

### 2.7 Immunofluorescence staining

Chondrocytes were treated with different drug treatments for 24 h on glass coverslips in 6‐well plate. After PBS washing, the chondrocytes were fixed and blocked before incubating with the primary antibody against Nrf2 (80593‐1‐RR, Proteintech) and NF‐κB (8242, cell signaling technology) overnight at 4°C. After that, the cells were washed and stained with Alexa Fluor 488 or Alexa Fluor 594 at room temperature. After 1 h incubation, the cells were then counterstained with DAPI for another 10 min. In the end, the slides were observed under a fluorescence microscope (BX53, OLYMPUS).

### 2.8 Western blotting

Cells were first lysed in radioimmunoprecipitation assay (RIPA) buffer to extract proteins. The protein levels were with a BCA protein assay kit. Samples containing 40 μg of protein were separated by 12% SDS‐PAGE, and then the proteins were transferred to PVDF membrane. The membrane was blocked with 5% skim milk for 2 h and then incubated with primary antibodies (1:1000), including GAPDH (60004‐1‐Ig, Proteintech), β‐actin (81115‐1‐RR, Proteintech), Nrf2 (80593‐1‐RR, Proteintech), NF‐κB (8242, cell signaling technology), collagen II (ab34712, Abcam) and MMP‐9 (ab76003, Abcam). After washing thrice for 5 min each in TBST, the membrane was incubated with secondary antibody conjugated with horseradish peroxidase (1:3000) for 1 h. After washing, the immunoreactive bands were visualized and quantified. GAPDH was used as an internal marker.

### 2.9 Quantitative real‐time PCR

Chondrocytes were treated with different drug treatments for 24 h on glass coverslips in 6‐well plate. Total RNA was extracted with Trizol and assessed by nanodrop method. The A260/A280 ratio was calculated for each sample, with the value between 1.6 and 2.0 considered as acceptable. Reverse transcription of RNA was conducted with Prime script RT reagent kit. Detailed primer information was listed in Table S1. qPCR was conducted with ex Taq SYBR‐green PCR (Takara) according to the manufacturer's instructions. The expression of β‐actin was used as a control to normalized the mRNA level of individual genes.

### 2.10 Coculture of Raw264.7 and chondrocytes

Rat chondrocytes and Raw264.7 macrophages were cocultured in a transwell system with 0.4 μm pore membrane insert. RAW 264.7 macrophages were seeded in the upper chamber, and rat chondrocytes were seeded in the 6‐well plate placed in the lower chamber. After 24 h culturing, RAW 264.7 macrophages were pretreated with LPS (1000 ng/mL) for 24 h. Then BR was added into the lower side for treatment. In the end, the total protein and mRNA were extracted from rat chondrocytes for Western blot and PCR assay, respectively.

### 2.11 Animal experiments

Anterior cruciate ligament transection (ACLT) surgery was utilized to develop OA animal model in SD rats (6 weeks old, 180 g–220 g) as previously reported.^28^ After successful modelling, the rats were randomly assigned into following groups (six mice in each group): Group 1: Healthy control group, received 0.9% saline injections intraarticularly for 8 weeks; Group 2: ACLT group, received 0.9% saline intraarticularly for 8 weeks; Group 3: ACLT + BR group, received BR (20 μM, 200 μL) intraarticularly for 8 weeks; Group 4: ACLT+HA group, received HA (50 mg/kg) intraarticularly for 8 weeks as positive control group. After the treatment, the animals were euthanized with knee joints collected for histopathological and immunohistochemical analyses.

### 2.12 Histological analysis

Knee joint tissues were collected, fixed and followed by decalcification for 4 weeks. Afterwards, the samples were embedded in paraffine and cut into 5‐μm‐thick sections. The cartilage tissues were collected and embedded with paraffin and cut into 5‐μm‐thick sections. The tissue slices were dewaxed, dehydrated before haematoxylin and eosin staining. For haematoxylin and eosin staining, the slice was stained with haematoxylin and eosin kit. For toluidine blue staining, the tissue slices were stained with toluidine blue staining kit according to the instructions (G3663, Solarbio). For safranin O staining, the slices were stained with safranin O staining kit according to the manufacturer's instruction (G1371, Solarbio). All the staining was observed under an optical microscope (BX53, OLYMPUS) and OARSI score was evaluated according to Hunter et al.^29^

The paraffin sections were conventionally dewaxed, then repaired the antigen, blocked endogenous peroxidase activity and blocked with 5% bovine serum albumin (BSA) for 1 h. The paraffin sections were then incubated with primary antibodies against COL2α (1:200) or TNF‐α (1:200) overnight at 4°C. On the second day, the samples were incubated with the appropriate secondary antibodies (HRP labelled) for 1 h at room temperature. After diaminobenzidine (DAB) chromogenic reaction and nucleus counterstaining, the slices were dehydrated and sealed. The COL2α and TNF‐α expression changes in cartilage tissues were observed under a microscope.

### 2.13 Statistical analysis

Statistical significance was determined by one‐way ANOVA followed by student's *t*‐test. Data were presented as mean ± SD. *p* < 0.05 was considered as statistically significant.

## 3 RESULTS

**FIGURE 1:**
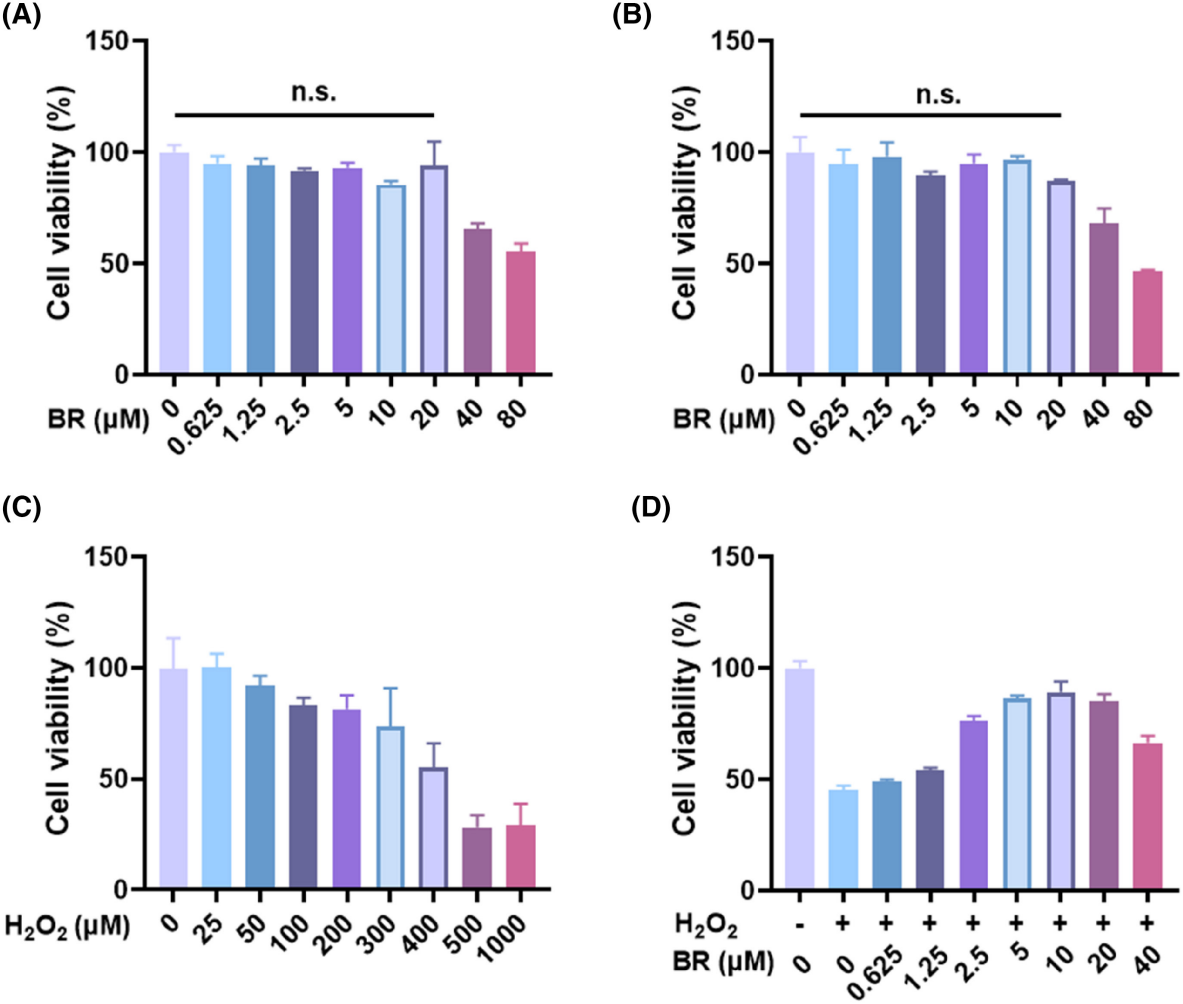
The chondrocyte viability after bilirubin treatment in the presence or absence of H_2_O_2_. The cell viability of rat chondrocytes after bilirubin treatment for (A) 24 h and (B) 48 h. (C) The viability of rat chondrocytes after exposure to different concentrations of H_2_O_2_ for 12 h. (D) The viability of rat chondrocytes after treatment with H_2_O_2_ (1000 μM) for 12 h, followed by incubation with different concentrations of BR for another 12 h. (mean ± SD, *N* = 3).

Previous animal studies have reported a wide effective therapeutic range for bilirubin (BR), but cell model studies have shown a narrow effective range of 10–20 μM for its therapeutic effects.^30^, ^31^, ^32^ To assess the in vitro cytotoxicity of BR in chondrocytes, the MTT assay was used, and the cells showed high survival rates ranging from 0.625 μM to 80 μM, with a survival rate of over 90% at concentrations below 40 μM (Figure 1A,B). The cell viability was about 60% at a concentration of 40 μM and 80 μM, indicating the overdosed BR had certain toxicity. It might be due to that overdosed BR was precipitated and therefore killed the cells. Oxidative stress plays a crucial role in the development and progression of OA.^33^ Hydrogen peroxide (H_2_O_2_) is a typical ROS elevated in the context of inflammation‐associated oxidative stress. To investigate the effects of H_2_O_2_ on rat chondrocytes, chondrocytes were exposed to different concentrations of H_2_O_2_ for various periods of time, which caused a visible decrease in cell viability compared to the untreated control group (Figure 1C). However, BR treatment could protect cells from H_2_O_2_ exposure in a dose‐dependent manner within 20 μM (Figure 1D). These results indicated that BR can be readily taken up by chondrocytes and is able to relieve ROS‐mediated oxidative stress as a potent antioxidant agent. What should be mentioned was that the overdosed BR showed cytotoxicity towards chondrocytes but exerted protective effect to certain extent in H_2_O_2_‐stimulated chondrocytes. These results indicated that BR was inclined to scavenge ROS firstly, therefore protecting chondrocytes from damage from oxidative stress.

**FIGURE 2:**
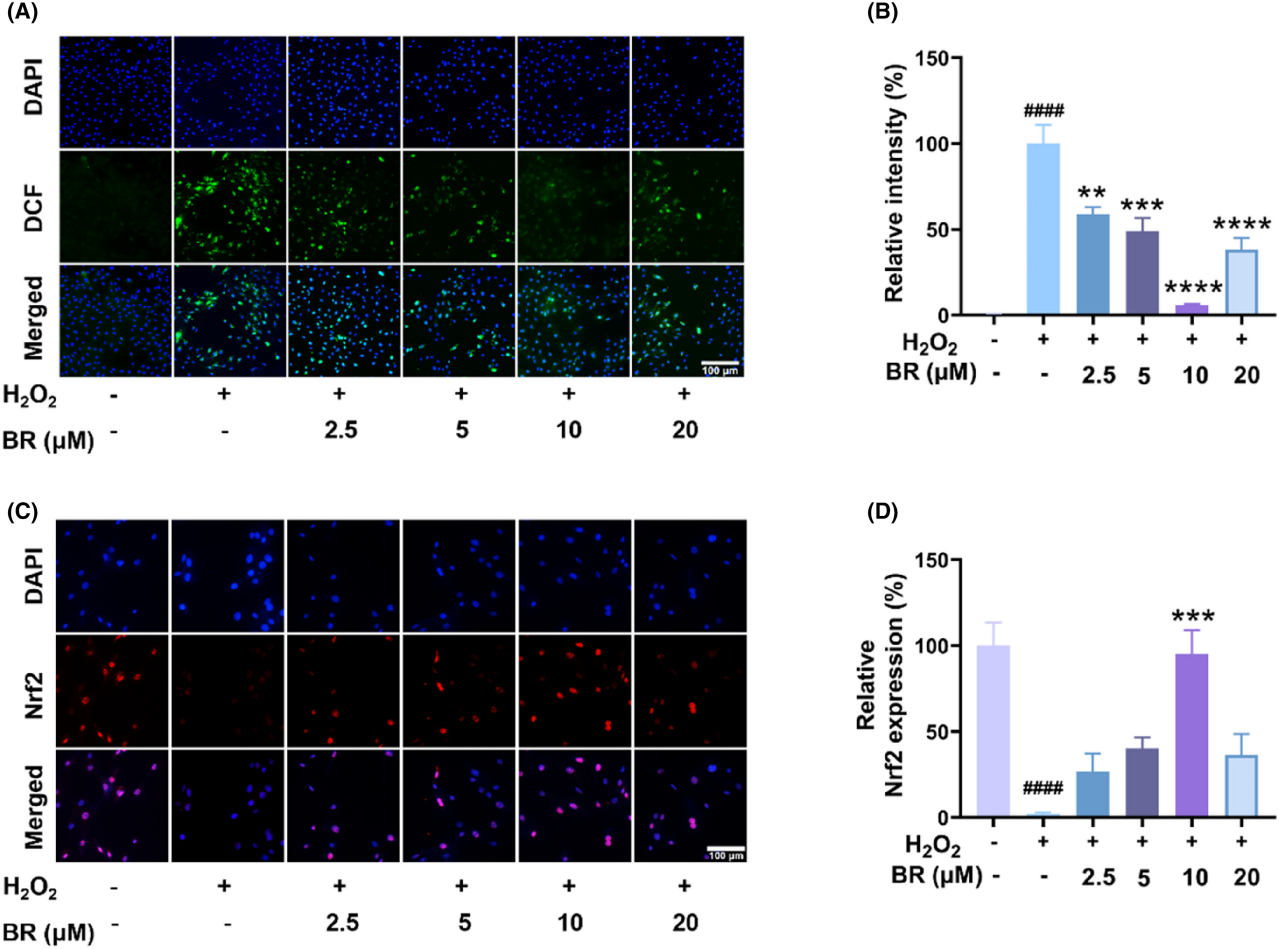
Bilirubin attenuates oxidative stress induce by H_2_O_2_ in chondrocytes. The rat chondrocytes were treated with different concentrations of bilirubin after exposure to H_2_O_2_ (1000 μM). (A) The represent images of chondrocytes stained with DCF to indicate the intracellular ROS. (B) Relative fluorescent intensity of the results in (A). (C) Representative images of immunofluorescence staining for nuclear Nrf2 in rat chondrocytes after bilirubin treatment and (D) quantitative analysis. (mean ± SD, *N* = 3). **p < 0.01, ***p < 0.001 and ****p < 0.0001 compared to the H_2_O_2_ group; ^####^ *p* < 0.0001 compared to the vehicle group.

To further investigate the effect of BR treatment on cellular oxidative stress, the level of ROS was measured in chondrocytes using the DCFH‐DA probe. As shown in Figure 2A,B, the relative DCF signal significantly increased after exposure to H_2_O_2_ for 12 h, but decreased after treatment with BR. These results strongly suggested that BR could effectively alleviate oxidative stress by scavenging ROS.

Activation of the Nrf2/HO‐1 system is a critical defence mechanism against oxidative stress. Under normal conditions, Nrf2 is bound to Keap1 in the cytoplasm and is degraded through ubiquitination. However, when cells are exposed to oxidative stress, Keap1 is oxidized and dissociates from Nrf2, allowing Nrf2 translocate into the nucleus where it can bind to the antioxidant response element and activate the transcription of various antioxidant genes, including HO‐1.^34^ This activation of the Nrf2/HO‐1 system is an important step in counteracting oxidative stress and preventing cell damage.^35^ However, overproduced ROS could impair the Nrf2/HO‐1 system and therefore damage/kill cells. To investigate the effect of BR on Nrf2 signalling in OA, immunofluorescence assay was performed. As presented in Figure 2C, Nrf2 nuclear translocation was occurred, evidenced by the cellular immunofluorescence staining. Our results revealed a dose‐dependent increase in Nrf2 nuclear translocation after treatment with BR (2.5–10 μM) (Figure 2C,D).

**FIGURE 3:**
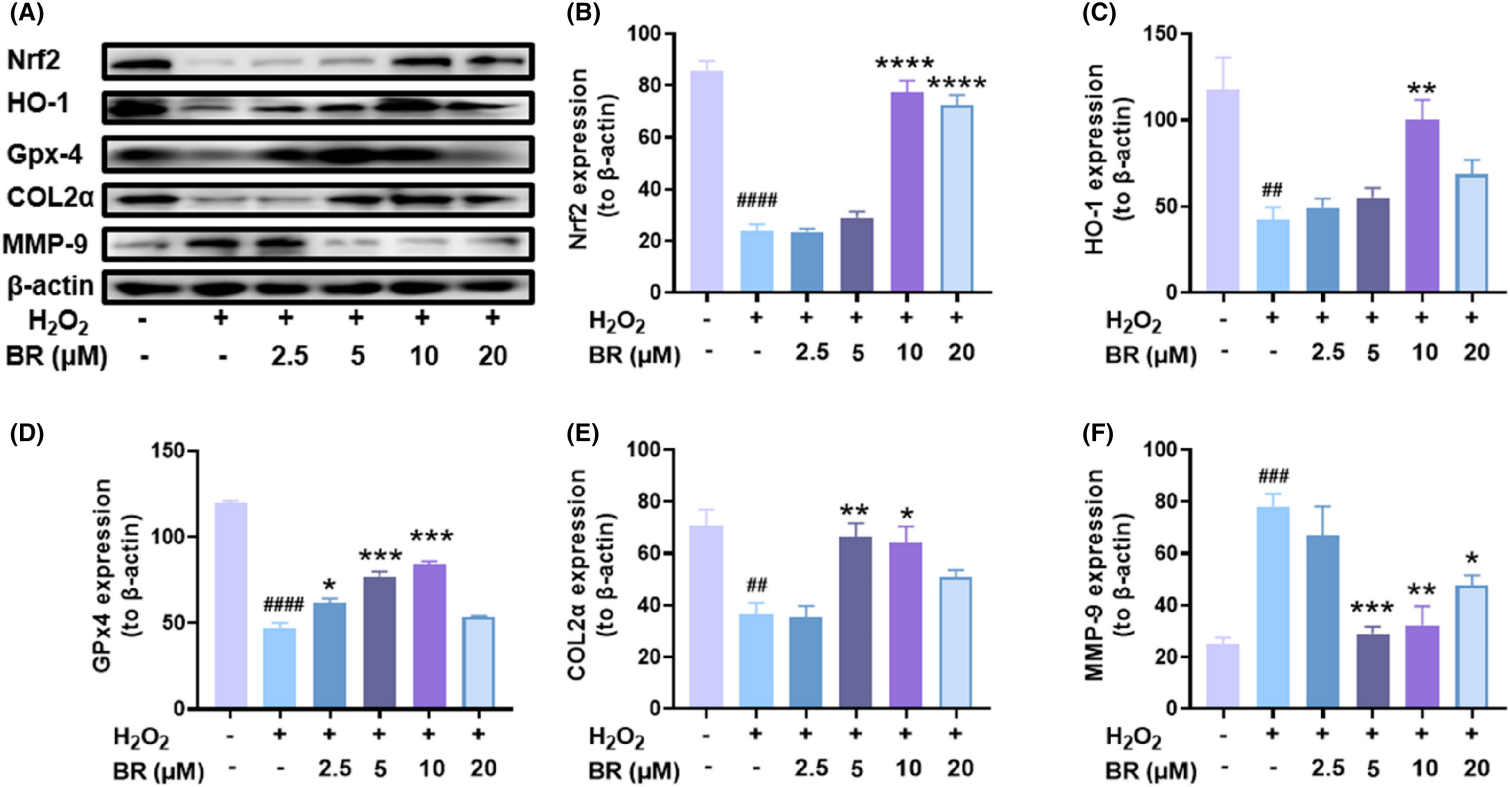
Bilirubin exerts chondroprotective effects by activating Nrf2/HO‐1 signalling pathway. The chondrocytes were treated with different concentrations of bilirubin after exposure to H_2_O_2_ (1000 μM). (A) Western blot analysis of protein expression of Nrf2, HO‐1, GPx4, collagen II and MMP‐9 in chondrocytes after various treatments. Quantitative analysis of (B) Nrf2, (C) HO‐1, (D) Gpx‐4, (E) Col2α and (F) MMP9 in the Western blot results. (mean ± SD, *N* = 3). *p < 0.05, **p < 0.01, ***p < 0.001 or ****p < 0.0001 indicates statistical difference compared to the H_2_O_2_ group, and ^####^ *p* < 0.0001 indicates statistical difference compared to the vehicle group.

**FIGURE 4:**
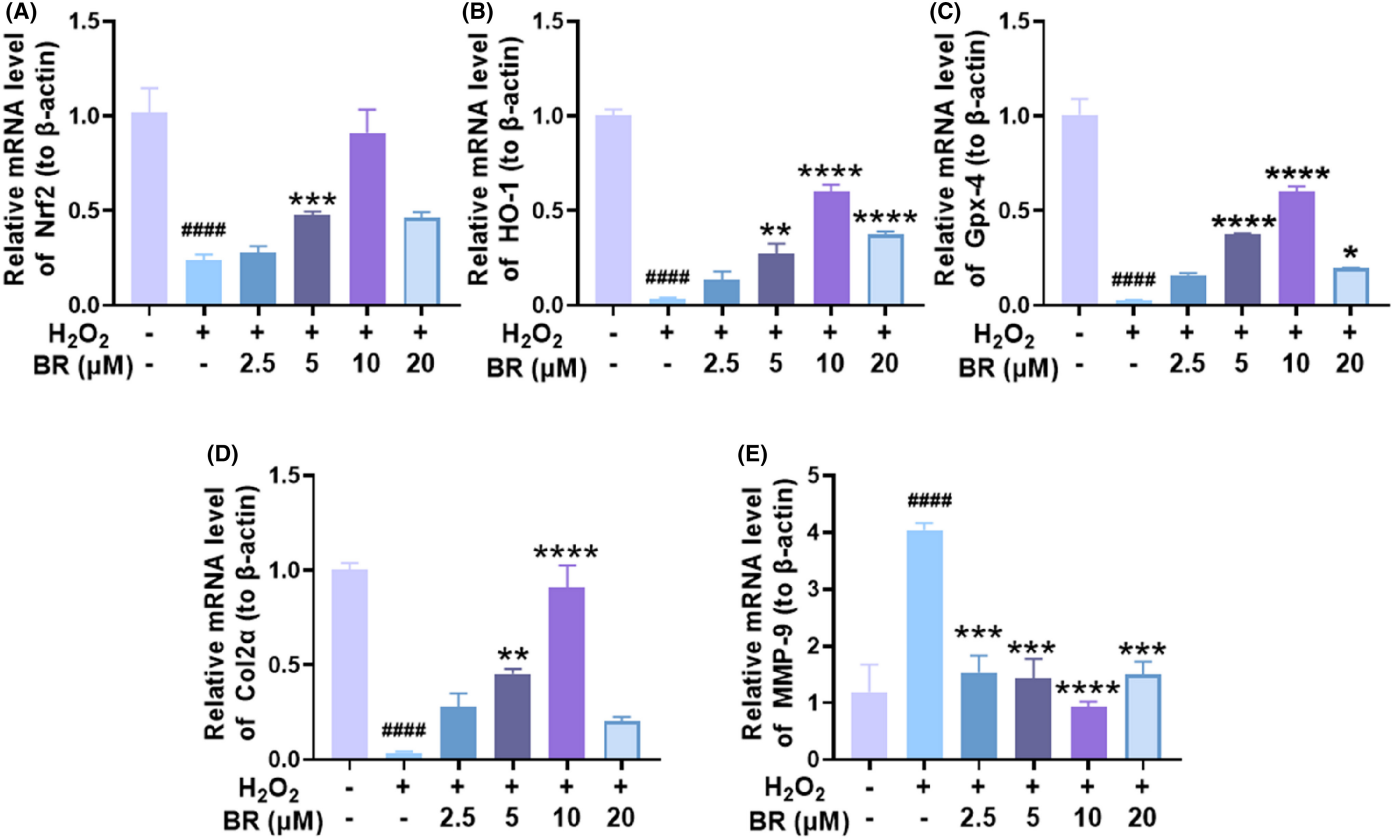
qRT‐PCR was performed to investigate the mRNA changes in chondrocytes after various treatments. The mRNA expression levels of (A) Nrf2, (B) HO‐1, (C) Gpx‐4, (D) Col2α and (E) MMP9 were determined by qRT‐PCR in H_2_O_2_ injured chondrocytes treated with BR under different concentrations. (mean ± SD, *N* = 3). *p < 0.05, **p < 0.01, ***p < 0.001 and ****p < 0.0001 compared to the H_2_O_2_ group; ^####^ *p* < 0.0001 compared to the vehicle group.

HO‐1 is a downstream target of the Nrf2 pathway.^36^ To further explore whether BR stimulates the Nrf2/HO‐1 signalling pathway, we then performed Western blot and q‐PCR to detect changes in Nrf2/HO‐1 protein and mRNA expression levels, respectively, after H_2_O_2_‐stimulated chondrocytes were incubated with BR for 12 h. As shown in Figure 3A–C, Nrf2 and HO‐1 expressions were impaired after incubation with H_2_O_2_, but dose‐dependently increased after treatment with BR, consistent with the immunofluorescence results. Moreover, the protein expression of oxidative stress‐related marker, GPx4, was also dramatically increased after treatment with BR in a dose‐dependent manner within 20 μM (Figure 3A,D). Collagen II is one of the major components of chondrocyte matrix and contributes to preserving cell phenotype, while MMP‐9 is a matrix catabolic enzyme that causes deleterious damage to cartilage. The relative level of catabolic indicator MMP‐9 was increased after exposure to H_2_O_2_ but decreased with BR treatment, while COL2α level showed the opposite trend (Figure 3E,F). Furthermore, the mRNA results demonstrated a prominent increase in Nrf2 (Figure 4A), HO‐1 (Figure 4B) and Gpx4 (Figure 4C), as well as collagen II (Figure 4D), but a decrease in MMP9 (Figure 4E). These results indicated that BR promotes Nrf2 nuclear translocation and activates the antioxidant system in rat chondrocytes, therefore suppressing the increased ROS level. In addition, the increased collagen II and decreased MMP9 confirmed that BR treatment was correcting the unbalanced metabolism of chondrocytes, beneficial for OA treatment.

**FIGURE 5:**
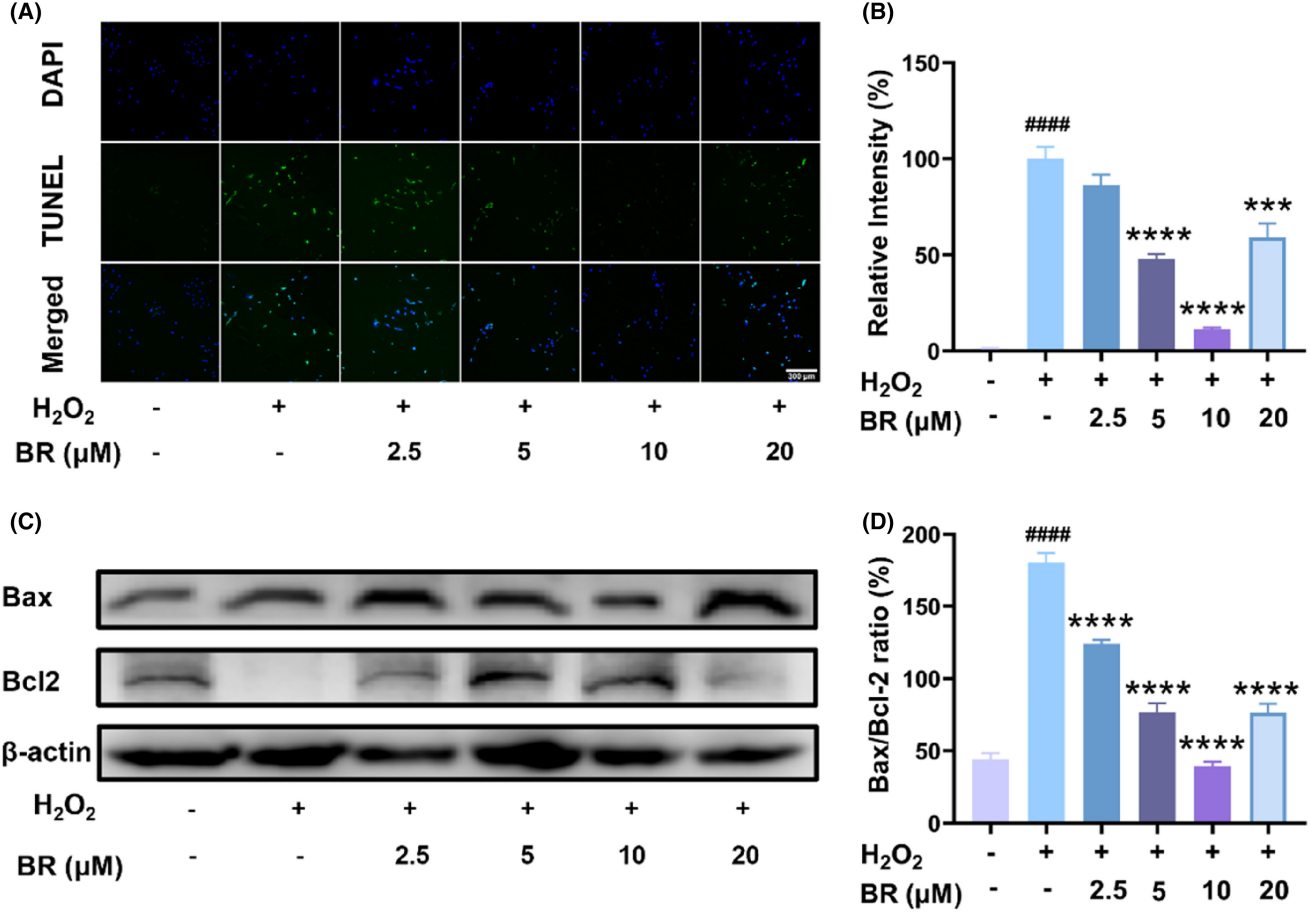
Bilirubin alleviated H_2_O_2_‐induced chondrocytes apoptosis. The rat chondrocytes were treated with different concentrations of bilirubin after exposure to H_2_O_2_ (1000 μM). (A) Representative TUNEL staining images of chondrocytes. (B) Quantified analysis of TUNEL assay. (C) The Western blotting results of Bax and Bcl‐2. (D) The ratio of Bax/Bcl‐2 levels calculated from Western blotting. The results were presented as the mean ± SD of three independent experiments (*N* = 3). ****p < 0.001 vs H_2_O_2_ group, ####*p* < 0.0001 versus vehicle group.

Chondrocytes are the only cells in the cartilage and are responsible for maintaining cartilage regeneration. Because articular cartilage is avascular and without cell turnover, the articular erosion degree is almost positively linked with the rate of chondrocyte apoptosis.^37^, ^38^ It has been reported that the apoptotic percentage of chondrocytes is higher in OA cartilage samples than that in normal cartilage (18%–21% vs. 2%–5%).^39^ Therefore, we further investigated the effect of BR on the apoptosis of H_2_O_2_‐treated chondrocytes in vitro. As shown in Figure 5, TUNEL staining and Western blotting were conducted to assess the antiapoptotic effect of bilirubion. 10 μM of BR showed a better antiapoptotic effect compared to the lower or higher dosages. Moreover, the increased ratio of Bax/Bcl2 after H_2_O_2_ exposure was suppressed by BR, and the effect displayed a dose‐dependent manner within 10 μM. Further increased dosage has a reverse effect. These results demonstrated that BR can exert protection against oxidative stress‐induced apoptosis in chondrocytes.

**FIGURE 6:**
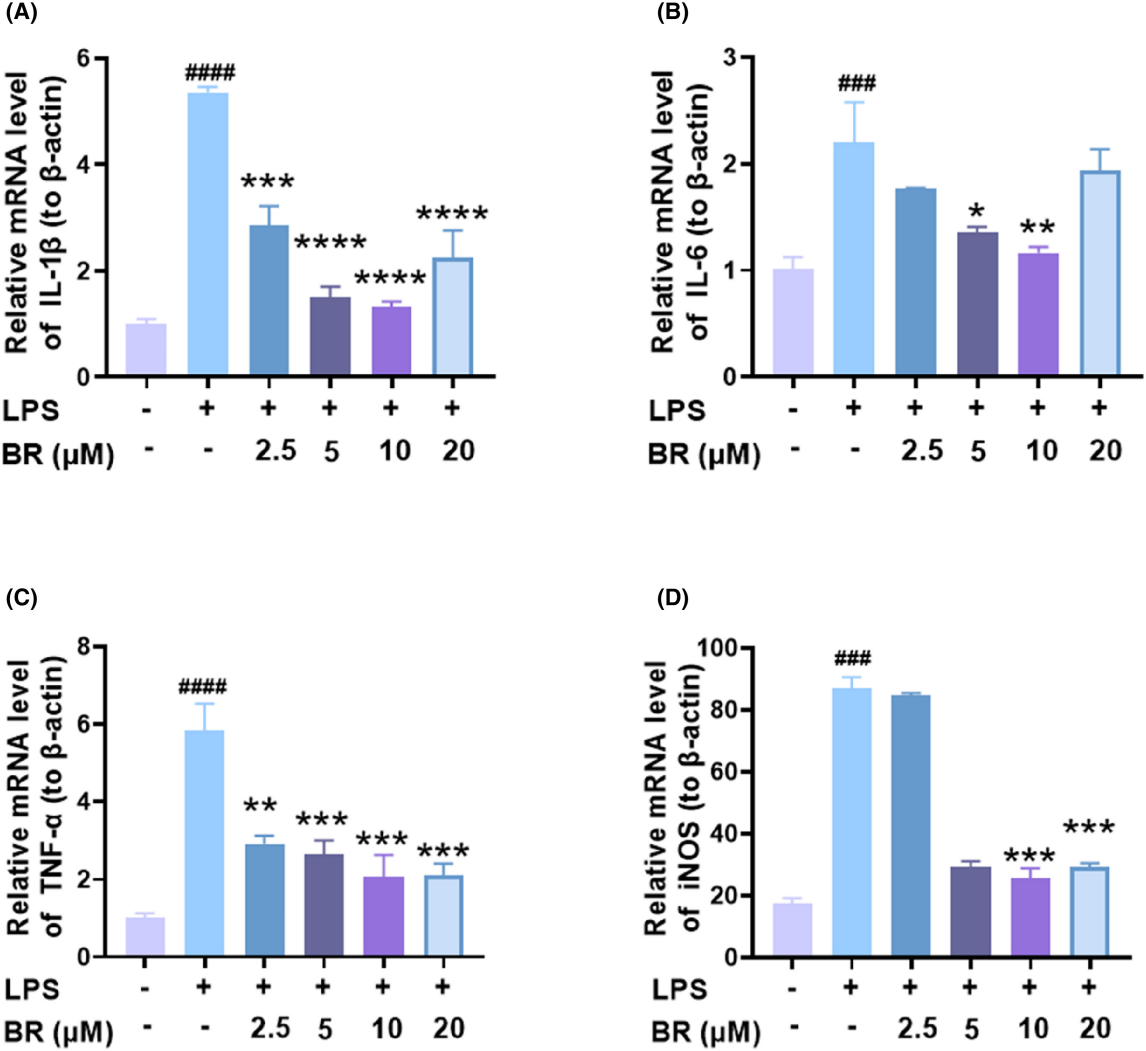
BR alleviates inflammation in LPS‐induced RAW264.7 cells. The mRNA levels of the inflammatory cytokines, including (A) IL‐1β, (B) IL‐6, (C) TNF‐α and (D) iNOS, were determined by qRT‐PCR. The internal control for normalization of gene expression was β‐Actin. (mean ± SD, *N* = 3). *p < 0.05, **p < 0.01, ***p < 0.001, and ****p < 0.0001, indicate the statistical difference compared to the H_2_O_2_ group; ^###^ *p* < 0.001 and ^####^ *p* < 0.0001 versus vehicle group.

OA is a chronic and degenerative disease that affects the articular cartilage of joints. Chronic systemic inflammation and oxidative stress are key players in the pathogenesis of OA. These factors can lead to cartilage tissue and cell damage, resulting in catabolic degradation of extracellular matrix (ECM) and loss of aggrecan and collagen II. In addition to the structural damage, OA is also characterized by low‐grade chronic systemic inflammation that can result from metabolic disturbances.^40^, ^41^ The migration of macrophages to the injured articular cartilage during OA progression can further aggravate inflammation and rapidly reduce chondrocyte function.^42^ Therefore, it was further investigated that whether BR can reduce the production of pro‐inflammatory cytokines in LPS‐stimulated macrophages. Raw264.7 cells were firsly stimulated with 1000 ng/mL of LPS to induce inflammation and then treated with BR in different concentrations. The mRNA expressions of TNF‐α, IL‐1β, IL‐6 and iNOS were analysed by qRT‐PCR (Figure 6). LPS stimulation could effectively elevated the mRNA levels of these pro‐inflammatory cytokines, indicating the inflammatory state formed. BR treatment significantly decreased the elevated inflammatory cytokines induced by LPS, and the trend was along with a dose‐dependent manner within 10 μM. Further increased dosage (20 μM) also showed considerable suppressing effect on TNF‐α and IL‐6 expression, but less effective on IL‐1β and iNOS compared to 10 μM, indicating that 10 μM might be the optimal concentration of BR for anti‐inflammation.

**FIGURE 7:**
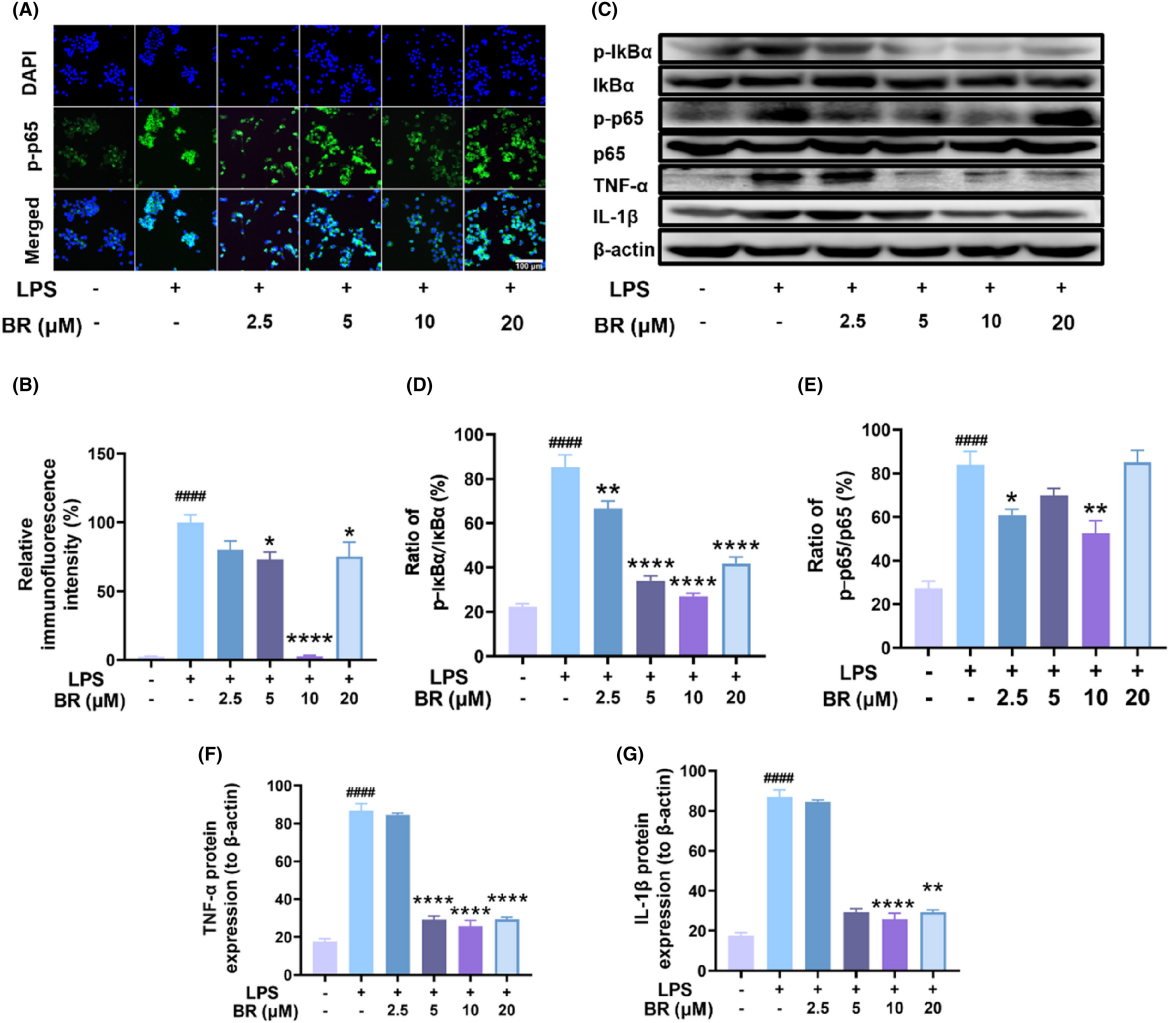
BR alleviates inflammation in LPS‐induced RAW264.7 cells by suppressing the NF‐κB signalling pathway. (A) Representative confocal images of phosphorylated p65 using immunofluorescent staining. Scale bar = 100 μM. (B) Quantitative analysis of fluorescence signal in (A). (C) Western blots of phosphorylated IκBα, total IκBα, phosphorylated p65, p65, TNF‐α and IL‐1β in LPS‐treated RAW264.7 cells. Statistical graphs of protein levels of (D) p‐IκBα/IκBα, (E) p‐p65/p65, (F) TNF‐α and (G) IL‐1β based on semi‐quantitate analysis of Western blot results. (mean ± SD, *N* = 3). *p < 0.05, **p < 0.01, ***p < 0.001 and ****p < 0.0001 versus H_2_O_2_ group, ^####^ *p* < 0.0001 versus vehicle group.

In particular, severe oxidative stress may cause inflammation through the activation of the NF‐κB pathway.^43^ NF‐κB, which is a primary regulatory factor in the expression of inflammation‐related cytokines and mediators, could be activated by LPS stimulation in macrophages.^13^, ^44^, ^45^ Therefore, we further investigated the effect of BR on NF‐κB activation. Immunofluorescent staining demonstrated that p65 phosphorylation levels were remarkably elevated by LPS stimulation, and BR significantly attenuated p65 phosphorylation in a dose‐dependent manner within 10 μM (Figure 7A,B). Similarly, even though 20 μM of BR also suppressed the p65 phosphorylation, the outcome was not effective as that of 10 μM of BR. Western blot analysis also showed that BR attenuated IκBα and NF‐κB phosphorylation in a dose‐dependent manner within 10 μM in activated macrophages (Figure 7C,D). In addition, the protein expressions of IL‐1β and TNF‐α were also suppressed by BR. These findings suggested that BR could attenuate the inflammatory responses in LPS‐pretreated macrophages by inhibiting NF‐κB signalling, also contributing to the OA treatment due to the important role of macrophage in OA inflammation.

**FIGURE 8:**
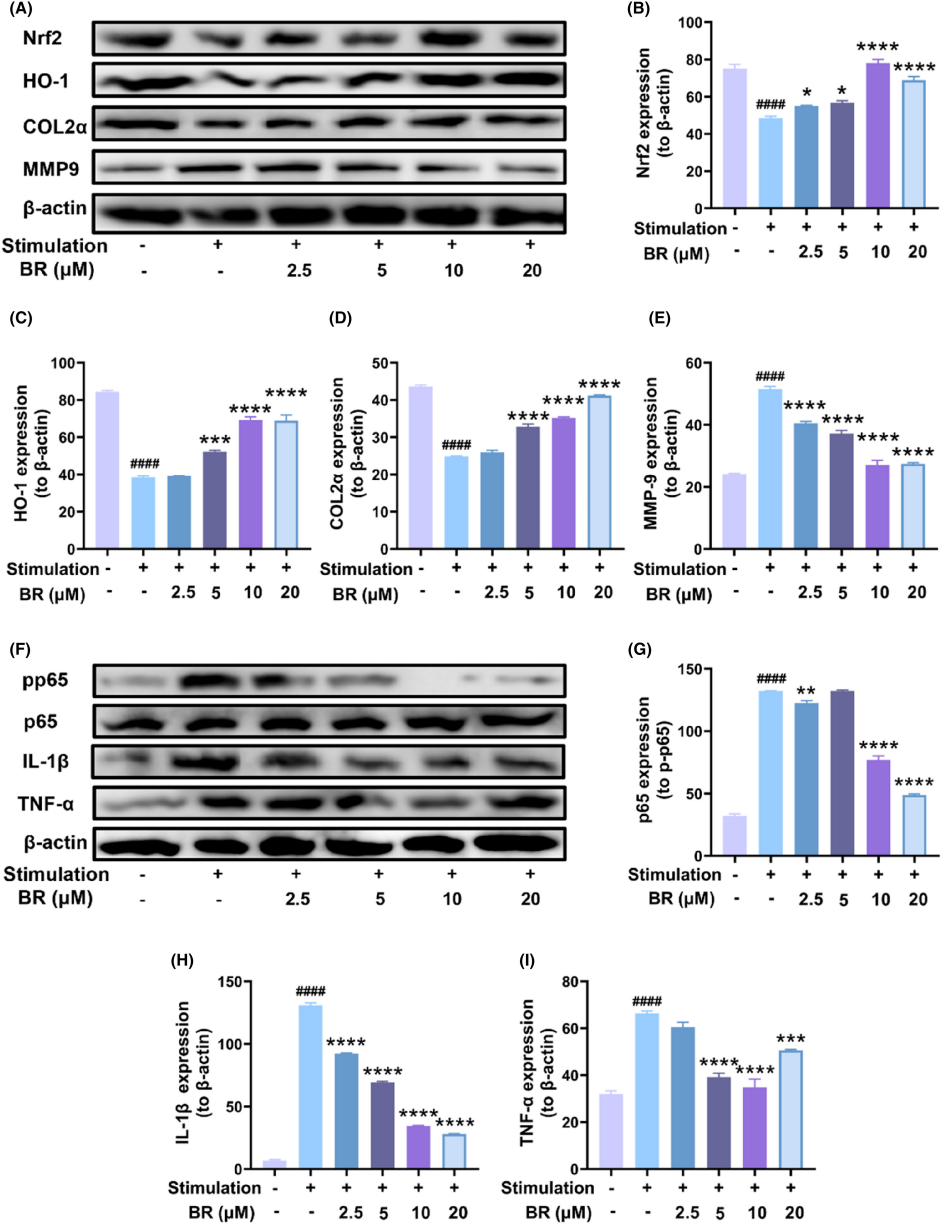
BR exerted antioxidant effects to protect chondrocytes when cocultured with LPS stimulated Raw264.7 cells. (A–E) Western blotting results and quantitative analysis of Collagen II, MMP‐9, Nrf2 and HO‐1 of chondrocytes cocultured with LPS‐stimulated Raw264.7 cells in the presence of BR (0, 2.5, 5, 10 and 20 μM). (F–I) Western blotting results and quantitative analysis of phosphorylated p65, total p65, IL‐1β and TNF‐α of chondrocytes cocultured with LPS‐stimulated RAW264.7 cells in the presence of BR (0, 2.5, 5, 10 and 20 μM). (mean ± SD, N = 3). *p < 0.05, **p < 0.01, ***p < 0.001 and ****p < 0.0001 versus H_2_O_2_ group; ^####^ *p* < 0.0001 versus vehicle group.

Following that, we further cocultured LPS‐stimulated RAW264.7 macrophages and chondrocytes in a transwell plate to mimic the microenvironment of OA, and then investigated the therapeutic efficacy of BR. The Raw264.7 was seeded in the upper side, and the chondrocytes were grown in the lower side. LPS was used to stimulate Raw264.7 to an inflammatory state, and the oxidative and inflammatory states of chondrocytes were evaluated with or without BR treatment. As shown in Figure S1, the inflammation state induced by LPS suppressed the components in the antioxidant system of chondrocytes, including HO‐1, Nrf‐2 and Gpx‐4. As expected, BR treatment recovered them to some extent, and the efficacy was dose dependent within 10 μM, similar as above results. Furthermore, we also monitored the protein expression of HO‐1 and Nrf2 in chondrocytes, and the results showed a similar trend as the PCR results (Figure 8A–C). These results suggested that BR could exert protective effect for chondrocytes in OA microenvironment by activating the Nrf2/HO‐1 signalling pathway.

In addition, we also tested the expression of inflammatory cytokines in the chondrocytes. As shown in Figure S2, the coculture of LPS‐stimulated Raw264.7 with chondrocytes upregulated the mRNA levels of pro‐inflammatory cytokines (IL‐1β, IL‐6 and TNF‐α) in chondrocytes, and BR treatment suppressed them to a certain exert. Western blot showed a similar trend for IL‐1β and TNF‐α. These results suggested that BR could alleviate the inflammation state of chondrocytes in OA microenvironment. Moreover, we tested the phospholation of p65 after various treatments (Figure 8F,G). It was shown that BR suppressed the activation of NF‐κB pathway induced by macrophages and also the expression of IL‐1β and TNF‐α (Figure 8H,I). Interestingly, 20 μM of BR did not show sufficient protective and anti‐inflammation effects compared to 10 μM of BR. This might be due to the insufficient solubility of BR, which resulted in erratic presentation and limited its effect in OA treatment. Overall, BR exert protect effect for chondrocytes in OA microenvironment by activating Nrf‐2/HO‐1 pathway and suppressing NF‐κB pathway. The therapeutic efficacy was also evidenced by the increased Collagen II level and decreased MMP‐9 level, which demonstrated a healthier state of chondrocytes.

**FIGURE 9:**
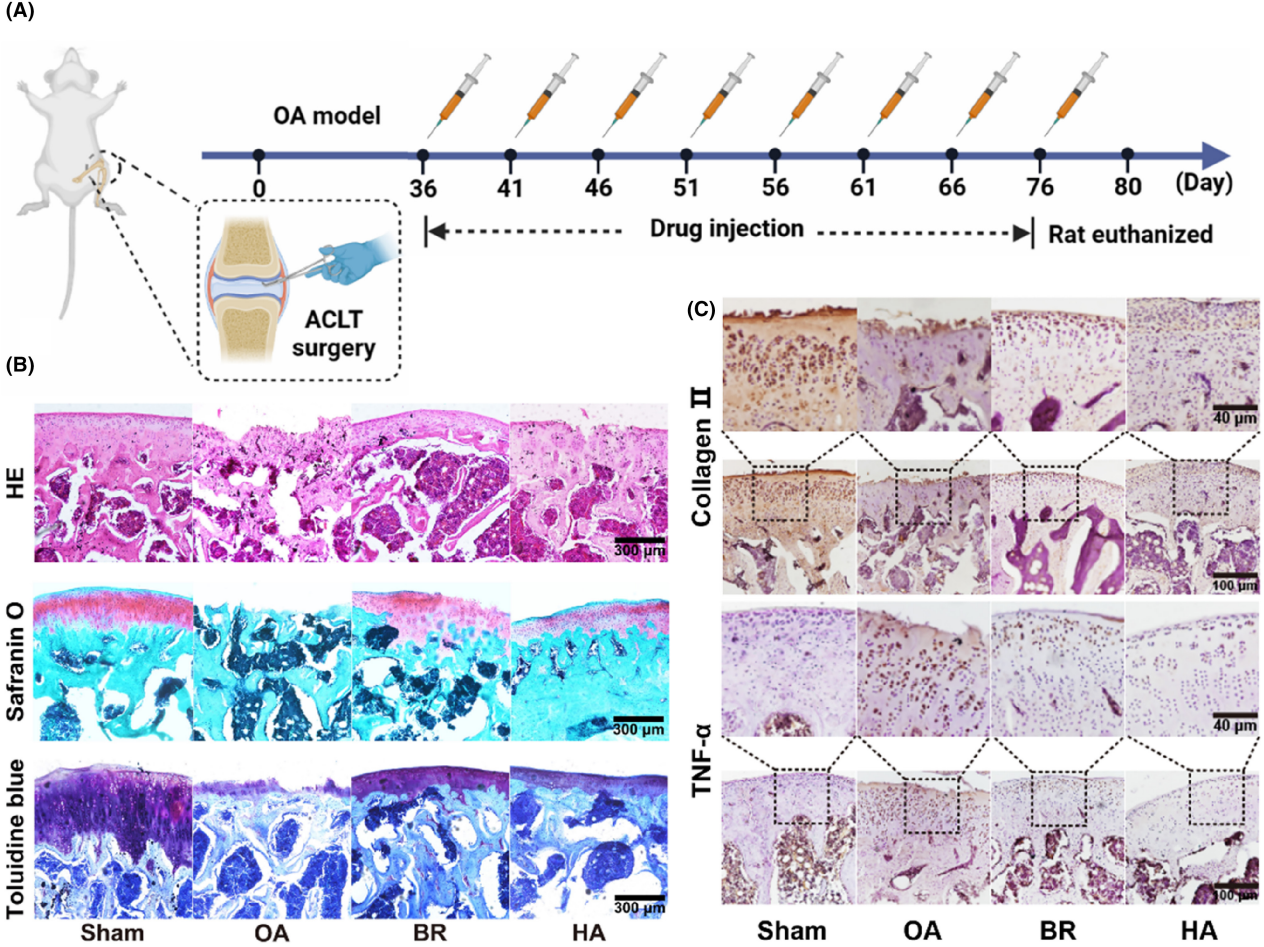
Bilirubin improved the articular cartilage injury of OA rats. (A) Schematic design of animal experiments. (B) Representative images under microscopy of normal group, OA group, BR treatment group and HA treatment group after HE, safranin O/fast green, and Toluidine Blue staining, respectively. Magnification, × 100. Scale bar, 100 μm. (C) Representative immunohistochemical images for Collagen II and TNF‐α. Magnification, × 100. Scale bar, 100 μm.

After confirming the potent protective effects of BR in vitro, we next evaluated its in vivo therapeutic efficacy on OA. HA which has been widely used in clinic to ameliorate OA progression^46^ was used as a control here. OA model rats were induced by using ACLT surgery, and BR was intra‐articularly administered every 5 days (Figure 9A). After eight rounds of treatment and rat euthanized, the knee joints were collected for further investigation. As shown in Figure 9B, histopathological analysis with HE, safranin O/fast green and Toluidine Blue staining were performed, and the results indicated that ACLT surgery caused clear superficial cartilage erosion and proteoglycan loss. After 8 weeks of BR treatment, the destructive effect of ACLT was partially alleviated, and the protection effect of BR was distinct. Haematoxylin and eosin staining showed that the OA cartilage was significantly damaged, and BR treatment exhibited obvious protection on the cartilage surface and appearance. The OARSI score system was used to indicate the OA severity (Figure S3A). The BR group hold significantly lower scores than OA group. The scores of HA group were lower than those of OA group, but higher than those of BR. Safranin O/fast green staining also suggested that collagen deposition was much less in OA rats than that in the BR treatment group (Figure 9B and Figure S3B), consistent with the haematoxylin and eosin results. Toluidine blue staining showed that aggrecan gradually faded away in the OA group but recovered after BR treatment (Figure 9B and Figure S3C). HA also exhibited protection for cartilage surface, collagen deposition and aggrecan, to some extent, but it was not effective than BR. These results suggested that both BR and HA exert protection for OA cartilage, and BR showed the superior efficacy than the clinical applied HA.

To further investigate the in vivo effect of BR on OA, immunohistochemical staining was performed to assess the expression levels of pro‐inflammatory cytokine, TNF‐α. As presented in Figure 9C, the TNF‐α was upregulated in OA group. However, treatment with BR suppressed the secretion of inflammatory cytokines, and quantitative analysis further confirmed that these interventions helped reduce the levels of inflammatory cytokines (Figure S4A). These results confirmed the anti‐inflammatory properties of BR. Collagen type II is one of the major components of the chondrocyte matrix and contributes to preserving cell phenotype.^47^ Therefore, we investigated the effect of BR on the abnormal degradation of collagen type II in rat cartilage. The results in Figure 9C and Figure S4B showed that the expression of collagen type II at the protein level was significantly decreased in OA rats, but treatment with BR inhibited this process at the protein level. HA showed certain effect to inhibit TNF‐α and preserve Collagen II, but the therapeutic outcome was less than BR. These immunohistochemical results were consistent with the above results. Furthermore, we conducted an immunofluorescence assay to monitor Nrf2 expression, aiming to elucidate the in vivo antioxidative effects of BR (Figure S5). Our findings revealed a decrease in Nrf2 levels within the OA model, while BR treatment notably elevated Nrf2 expression. Notably, HA treatment also led to an increase in Nrf2 expression, albeit less effectively compared to BR. These outcomes distinctly illustrate BR's capacity to exert antioxidative properties in the OA rat model, thereby offering protective effects. Thus, these findings proved that BR could effectively halt the progression of bone damage and simultaneously repair bone erosion in a rat model of OA.

## 4 DISCUSSION

Osteoarthritis (OA) is believed to be caused by oxidative stress resulting from ROS accumulation and chronic inflammation.^48^ The excessive production of ROS inhibits the synthesis of type II collagen, destroys the extracellular matrix and causes the degeneration of cartilage tissue, which facilitates the progression of OA.^6^, ^15^ Antioxidant and anti‐inflammatory drugs have emerged as groundbreaking treatments that effectively slow down the OA progression.

Bilirubin (BR), a yellow bile pigment and the final metabolite of the heme catabolic pathway, was once considered a potentially harmful substance that indicates jaundice. However, it is actually a potent endogenous antioxidant that can scavenge various ROS, protecting cells and the body from oxidative stress‐mediated damage.^19^, ^49^, ^50^, ^51^, ^52^, ^53^, ^54^ BR binds to multiple cellular targets and transduces cellular signalling during metabolic homeostasis, and numerous studies have reported its antioxidant and anti‐inflammatory effects on inflammatory diseases.^50^, ^51^ Nonetheless, the therapeutic effect of BR on OA has not yet been tested, and the precise underlying mechanisms are still unclear. The primary objective of this study was to investigate whether BR exert protection on OA cartilage and the underlying mechanisms.

Excessive ROS production induced by H_2_O_2_ leads to oxidative stress and chondrocyte apoptosis. Therefore, we used H_2_O_2_ as an external stimulus to establish an injury model on chondrocytes. BR exerted considerable therapeutic effect to protect chondrocytes from H_2_O_2_‐inducd oxidative injury, and the in vitro optimal concentration is 10 μM. Of course, the results of the MTT assay indicated that up to 80 μM of BR were not severely toxic when cultured with chondrocytes. Western blot results and TUNEL staining showed a decrease in apoptotic cells in the H_2_O_2_‐induced chondrocyte model after BR treatment. The Bax/Bcl‐2 ratio was downregulated after BR treatment, further demonstrating the specific suppression of chondrocyte apoptosis by BR. Based on these results, it could be concluded that BR did exert protection on chondrocytes from H_2_O_2_‐inducd oxidative injury.

It is widely accepted that Nrf2 is critical in adaptive cellular responses to oxidative stress. Previous research has proved that the activation of Nrf2‐ARE pathway provides protection against H_2_O_2_‐induced cell death.^55^ In response to attacks by external or internal oxidants, Nrf2 is translocated from the cytoplasm to the nucleus, where it increases the transcription of antioxidant enzymes, including HO‐1.^34^ The Nrf2 pathway is the primary defence mechanism against oxidative stress and can be a promising target for combating diseases. After treated with BR, H_2_O_2_‐injured chondrocytes showed increased Nrf2 nuclear accumulation and elevated expression of HO‐1 and Gpx4, suggesting the activation of the Nrf2 pathway in BR‐treated chondrocytes. These results demonstrated that BR protects chondrocytes against oxidative stress by activating the Nrf2 pathway and upregulation of downstream Nrf2, HO‐1 and Gpx4.

**FIGURE 10:**
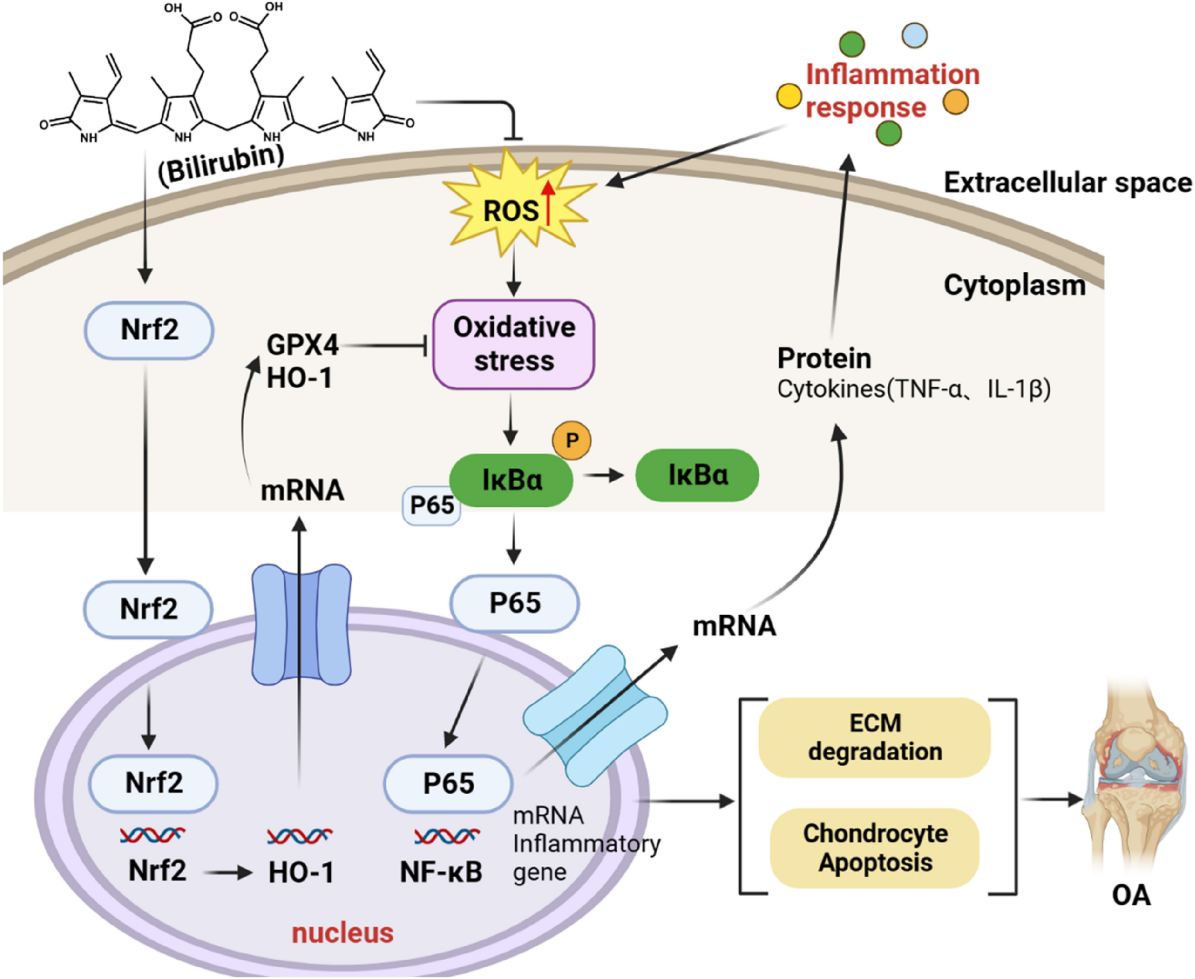
Schematic illustration of Bilirubin alleviating pathological and physiological progression of OA by relieving oxidative stress and reducing inflammation. Bilirubin has the ability to reduce oxidative stress by not only directly depleting radicals but also activating the Nrf2 pathway, as well we to decrease inflammation by suppressing NF‐κB pathway.

The pathogenesis and inflammatory response of OA are largely attributed to the stimulation of multiple interconnected immune‐signalling pathways and an imbalance in cytokine production. NF‐κB is an important factor in the inflammatory immune response and plays a significant role in the OA pathogenesis by regulating pro‐inflammatory mediators through phosphorylation. The activation of the NF‐κB signalling pathway could increase the expression of MMPs and initiate the release of inflammatory factors, thereby accelerating OA progression. Under physiological conditions, NF‐κB remains inactive in cytoplasm and binds to its inhibitory protein (IκBα). However, after stimulation by the OA microenvironment, phosphorylated NF‐κB translocates to the nucleus, associates with DNA and upregulates the expression of inflammation‐related genes such as IL‐1β and TNF‐α. This promotes ECM degradation in cartilage and chondrocyte apoptosis in OA. BR treatment reduced the protein levels of p‐p65, p‐IκBα and IκBα to the normal levels, indicating that BR specifically reduced the activation of the NF‐κB pathway, which is implicated in inflammation. Therefore, BR could also alleviate the inflammation in OA by suppressing NF‐κB pathway, besides its potent effect against oxidative stress (Figure 10).

In addition, we also investigated the in vivo protective effect of BR on cartilage degradation and the amelioration of OA progression, using the ACLT‐induced experimental OA rat model. BR significantly reduced cartilage degeneration by weekly intra‐articular injection in ACLT OA model, evidenced by the less severe cartilage degradation and milder structural alterations in the treatment group in morphological and histological assessments. Immunohistochemical staining revealed that the levels of TNF‐α and IL1β were increased in the OA group but decreased after treatment with BR. TNF‐α and IL1β are important inflammatory factors that alter the microenvironment around the injured tissue. In Safranin O fast green staining results, it was clear that BR treatment retained the morphology and extracellular matrix of cartilage compared to the OA model, demonstrating the protective property of BR for ACLT‐induced OA. Immunohistochemical staining was performed to assess the expression levels of type II collagen, and the analysis revealed that BR treatment increased type II collagen level, suggesting the ACLT‐induced OA progression was slowed. Our in vivo results are consistent with the in vitro effects of BR. We also included hyaluronic acid as a positive control and found that BR exhibited better therapeutic efficacy than the HA treatment group. Overall, our findings confirm the potent therapeutic efficacy of BR for OA treatment.

In summary, our study demonstrated that BR could significantly suppress the activated oxidative stress and increased apoptosis induced by H_2_O_2_, accompanied by the upregulation of antioxidant components, Nrf2 and HO‐1, as well as ameliorate LPS‐mediated inflammation in RAW264.7 cells by inhibiting the NF‐κB signalling pathway and the downstream pro‐inflammatory cytokines at the protein and mRNA levels. In the mimicking OA microenvironment, BR showed potent effect to protect chondrocytes. In an ACLT‐induced OA model, BR effectively reduced cartilage degeneration and delayed OA progression, evidenced by the retained collagen II and aggrecan. In the end, this study provides evidence for the first time that BR could be a potential therapeutic drug for OA by alleviating oxidative stress, suppressing inflammation and protecting chondrocytes from apoptosis.

## AUTHOR CONTRIBUTIONS

**Xinyu zhao:** Data curation (lead); investigation (lead); writing – original draft (equal). **Baiqun Duan:** Data curation (equal); investigation (equal); writing – original draft (equal). **Jianing Wu:** Investigation (supporting); methodology (lead). **Lihui Huang:** Methodology (equal); resources (lead). **Sheng Dai:** Resources (equal); software (lead). **Jie Ding:** Resources (supporting); software (supporting). **Meng Sun:** Software (equal); validation (lead). **Xinlu Lin:** Software (supporting); validation (supporting). **Yiling Jiang:** Validation (equal); visualization (equal). **Tuyue Sun:** Validation (supporting); visualization (supporting). **Ruijie Lu:** Methodology (equal); visualization (equal). **Huirong Huang:** Methodology (equal); project administration (equal). **Guangyong Lin:** Supervision (equal). **Ruijie Chen:** Supervision (equal). **Qing Yao:** Supervision (equal); writing – review and editing (equal). **Longfa Kou:** Conceptualization (equal); supervision (equal); writing – review and editing (lead).

## CONFLICT OF INTEREST STATEMENT

The authors confirm that there are no conflicts of interest.

## Supporting information


Appendix S1

## Data Availability

The data and materials which support the findings of this study are available from the corresponding author upon reasonable request.
