# Supplementary material for: Bilirubin ameliorates osteoarthritis via activating Nrf2/HO‐1 pathway and suppressing NF‐κB signalling
Source: J Cell Mol Med. 2024 Mar 17;28(7):e18173. doi: 10.1111/jcmm.18173 (PMC10945086; doi:10.1111/jcmm.18173)
Supplement: Supplementary file 1 — Appendix S1 [file JCMM-28-e18173-s001.docx]

**Table S1. Sequences of primers used in this study.**

| gene | Primer sequence (5’ to 3’) | Primer sequence (3’ to 5’) |
| --- | --- | --- |
| Rat collagen Ⅱ | GAGTGGAAGAGCGGAGACTACTG | GTCTCCATGTTGCAGAAGACTTTCA |
| Rat MMP-9 | AAGGGTACAGCCTGTTCCTGGT | CTGGATGCCGTCTATGTCGTCT |
| Rat MMP-13 | GGCTCCGAAATGCAGTCTTTCTT | ATCAAATGGGTAGAAGTCGCCATGC |
| Rat ACAN | AGGATGGCTTCCACCAGTGTGT | GGCATAAAAGACCTCACCCTCC |
| Rat Nrf2 | TCACACGAGATGAGCTTAGGGCA | TACAGTTCTGGGCGGCGACTTTAT |
| Rat HO-1 | CCCAAAACTGGCCTGTAAAA | CGTGGTCAGTCAACATGGAT |
| Rat Gpx4 | GGCTTCGTGTGCATCGTCACC | TTCACCACGCAGCCGTTCTTG |
| Mouse β-actin | GGCCAACCGTGAAAAGATGA | GACCAGAGGCATACAGGGACAA |
| Mouse iNOS | TCCCAGCCТGCCCCТТCA | CТCCТGCССАСТТССТСС |
| Mouse TNF-α | CAGGCGGTGCTTGTTCCTCAG | CGATGCGGCTGATGGTGTGG |
| Mouse IL1β | TCCGACCACCACTACAGCAAGG | GGAGCGTGCAGTTCAGTGATCG |
| Mouse IL-6 | GGTGTTGCCTGCTGCCTTCC | GCTCTGGCTTGTTCCTCACTACTC |


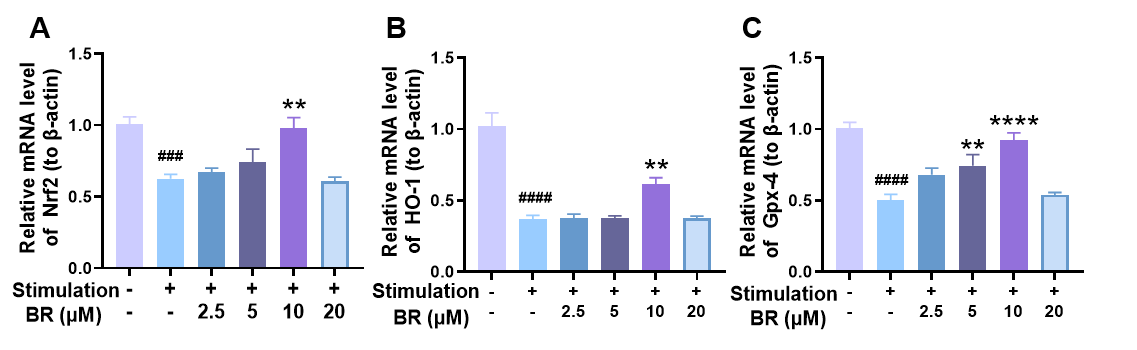


Figure S1. The mRNA levels of (A) Nrf2, (B) HO-1, and (C) Gpx-4 in rat chondrocytes after co-culture with LPS stimulated RAW264.7 cells.The results were presented as the mean ± SD of three independent experiments(N=3). * *P*<0.05 vs H_2_O_2_ group ** *P*<0.01 vs H_2_O_2_ group *** *P*<0.001 vs H_2_O_2_ group, **** *P*<0.0001 vs H_2_O_2_ group, #### *P*<0.0001 vs vehicle group, and statistical significance was determined by one-way ANOVA.


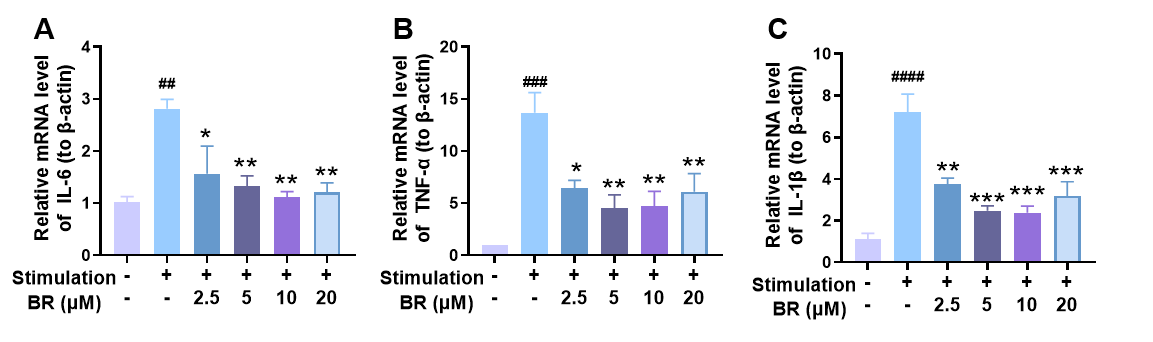


Figure S2. The mRNA levels of (A) IL-6, (B) TNF-α, and (C) IL-1β in rat chondrocytes after co-culture with LPS stimulated RAW264.7 cells. The results were presented as the mean ± SD of three independent experiments(N=3). * *P*<0.05 vs H_2_O_2_ group ** *P*<0.01 vs H_2_O_2_ group *** *P*<0.001 vs H_2_O_2_ group, **** *P*<0.0001 vs H_2_O_2_ group, #### *P*<0.0001 vs vehicle group, and statistical significance was determined by one-way ANOVA.


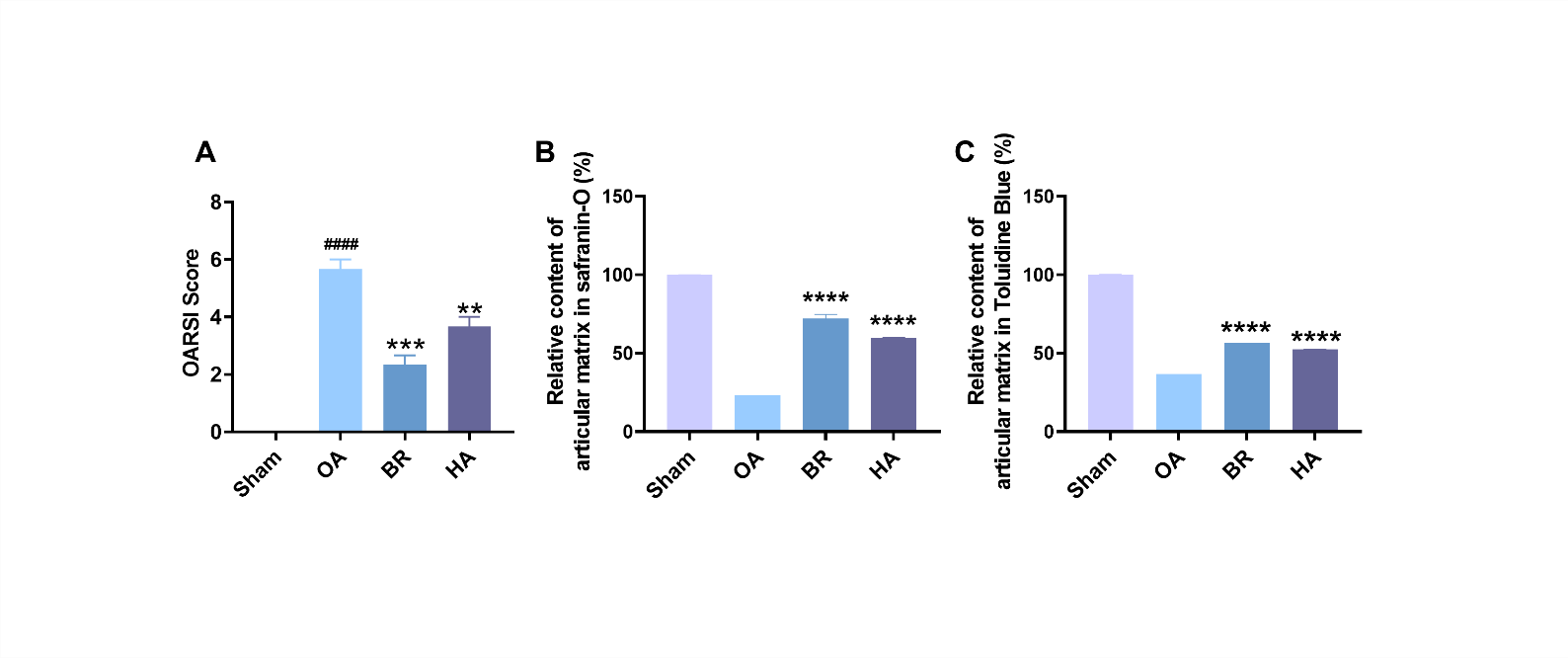


Figure S3. (A) The osteophyte osteoarthritis research society international (OARSI) score in rats after various treatments (N=6). Quantitative analysis of articular matrix in (B) safranin-O and (C) Toluidine Blue Staining (N=3). The results were presented as the mean ± SD. **P*<0.05 vs OA group, ** *P*<0.01 vs OA group, *** *P*<0.001 vs OA group, **** *P*<0.0001 vs OA group, ^####^ *P*<0.0001 vs Sham group.


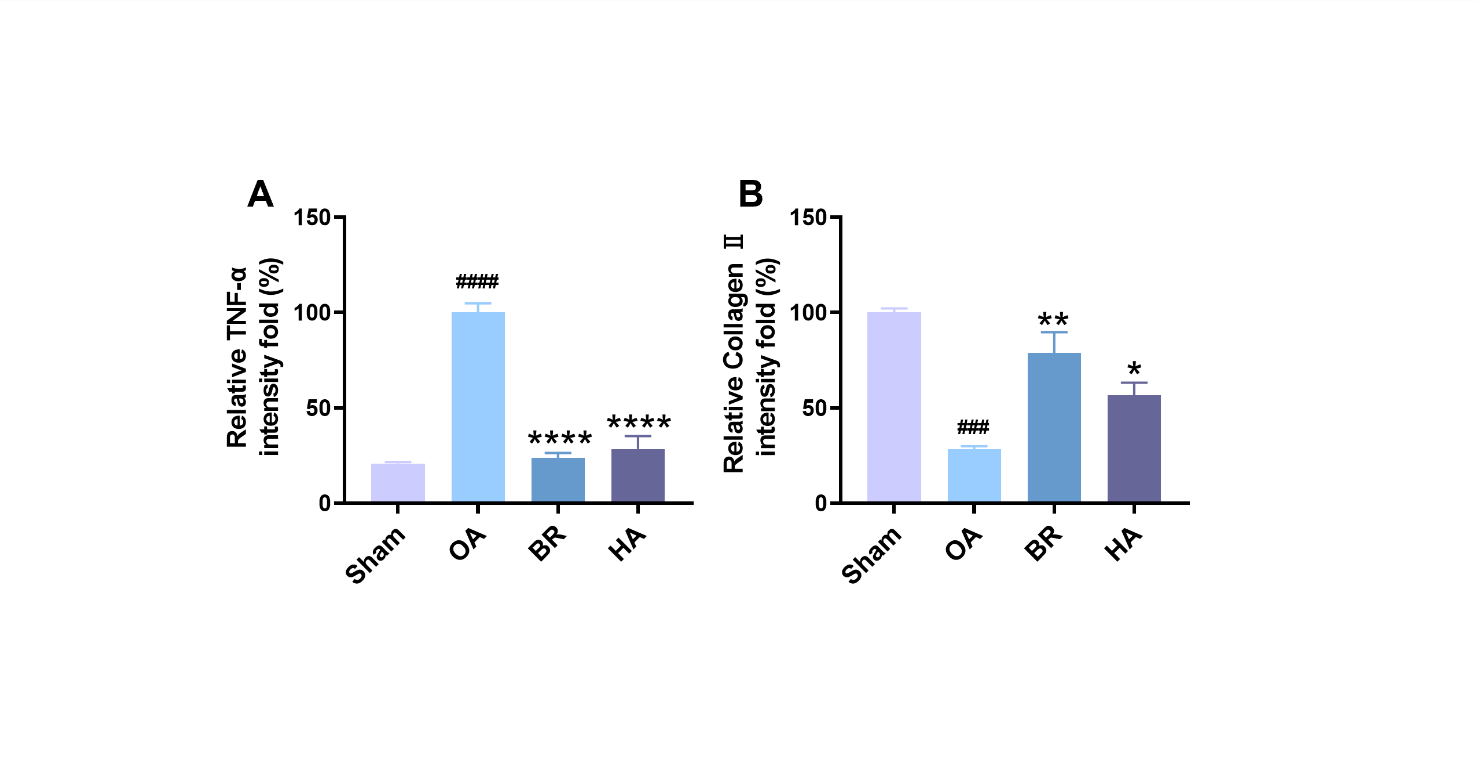


Figure S4. Quantitative analysis of (A) TNF-α and (B) Collagen II (N=3). The results were presented as the mean ± SD. * *P*<0.05 vs OA group, ** *P*<0.01 vs OA group, *** *P*<0.001 vs OA group, **** *P*<0.0001 vs OA group, ^####^ *P*<0.0001 vs Sham group.


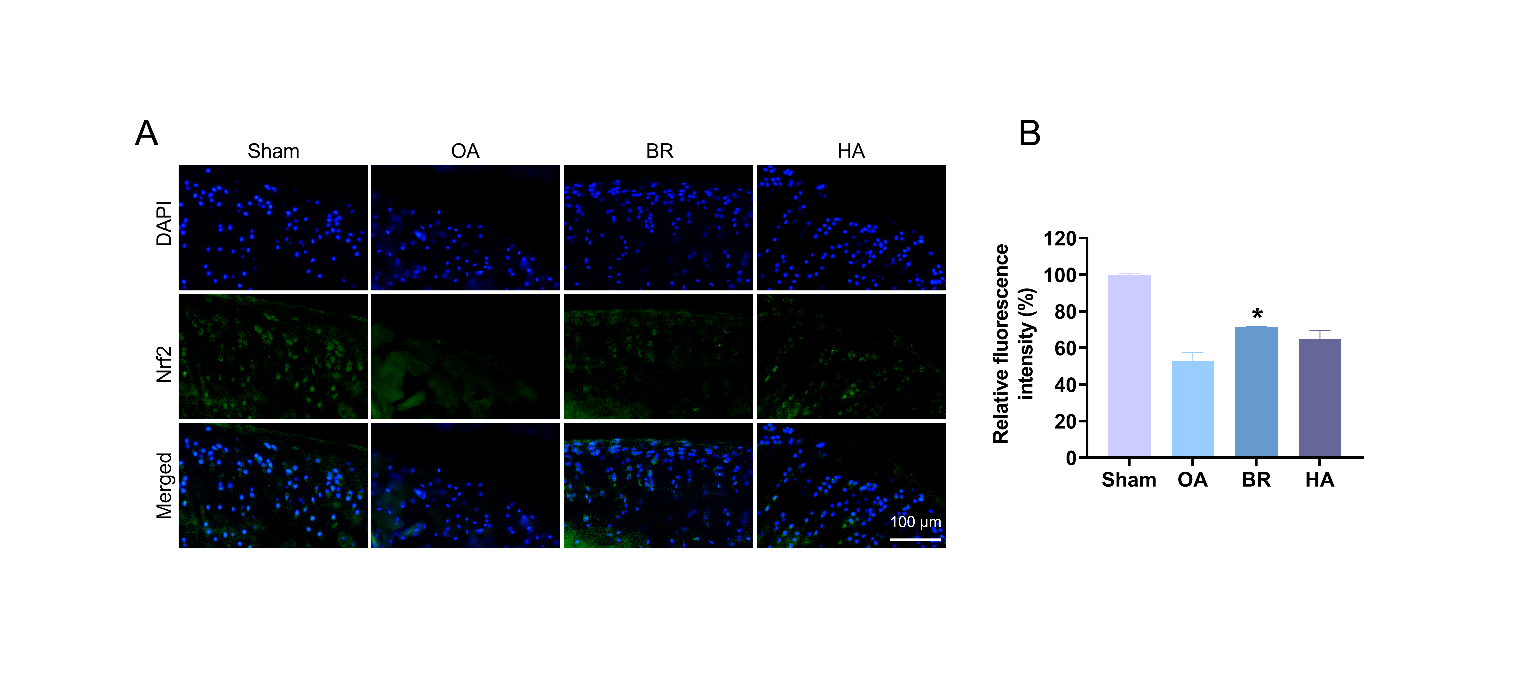


Figure S5. (A) Representative immunofluorescence images for Nrf2. Magnification, × 100. Scale bar, 100μm. (B) Quantitative analysis. * P<0.05 vs OA group.
